# Regulation of ribosomal RNA expression across the lifespan is fine-tuned by maternal diet before implantation

**DOI:** 10.1016/j.bbagrm.2016.04.001

**Published:** 2016-07

**Authors:** Oleg Denisenko, Emma S. Lucas, Congshan Sun, Adam J. Watkins, Daniel Mar, Karol Bomsztyk, Tom P. Fleming

**Affiliations:** a Department of Medicine, University of Washington, 850 Republican St., Rm 242, Seattle, WA 98109, USA; b Centre for Biological Sciences, University of Southampton, Mailpoint 840, Level D Lab & Path Block, Southampton General Hospital, Tremona Road, Southampton SO16 6YD, UK

**Keywords:** rDNA transcription, Maternal diet, DNA methylation, RRN3, TIF-1A, Thrifty gene

## Abstract

Cells and organisms respond to nutrient deprivation by decreasing global rates of transcription, translation and DNA replication. To what extent such changes can be reversed is largely unknown. We examined the effect of maternal dietary restriction on RNA synthesis in the offspring. Low protein diet fed either throughout gestation or for the preimplantation period alone reduced cellular RNA content across fetal somatic tissues during challenge and increased it beyond controls in fetal and adult tissues after challenge release. Changes in transcription of ribosomal RNA, the major component of cellular RNA, were responsible for this phenotype as evidenced by matching alterations in RNA polymerase I density and DNA methylation at ribosomal DNA loci. Cellular levels of the ribosomal transcription factor Rrn3 mirrored the rRNA expression pattern. In cell culture experiments, Rrn3 overexpression reduced rDNA methylation and increased rRNA expression; the converse occurred after inhibition of Rrn3 activity. These observations define novel mechanism where poor nutrition before implantation irreversibly alters basal rates of rRNA transcription thereafter in a process mediated by rDNA methylation and Rrn3 factor.

## Highlights

• Maternal malnutrition downregulates rDNA transcription in fetal tissues.
• Switch to normal diet permanently upregulates rDNA transcription compared to controls.
• These changes are mediated by DNA methylation and Pol I transcription factor Rrn3.
• This mechanism is activated before implantation.

## Graphical Abstract

**Figure:**
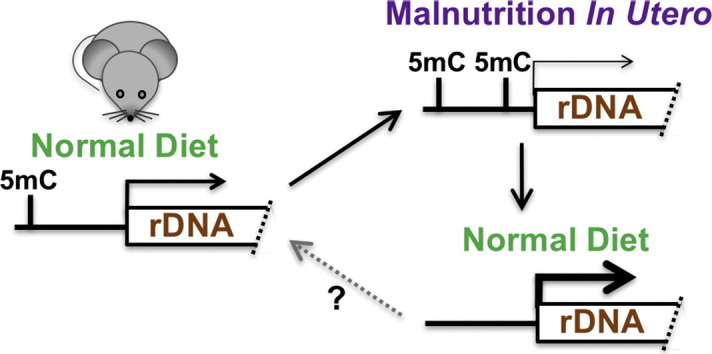

## 1 Introduction

Nutrient availability is one of the factors that limit rates of cell transcription, growth and division. Extensive studies in animal models and epidemiological evidence from across world populations have demonstrated that early environment, particularly poor nutrition in utero, is a risk factor for chronic diseases in adulthood [1], [2], indicating that permanent changes in transcription might be involved. A common phenotype in these studies is the combination of low birth weight (LBW, an index of poor fetal growth) with excessive ‘catch-up’ growth during early postnatal life. A conceptual framework for adverse developmental programming to account for these early markers is the ‘thrifty phenotype’ hypothesis. Here, poor maternal conditions would cause fetal adaptations to restrict growth and conserve energy for developmental needs. However, these responses would become maladaptive if nutrient levels improved postnatally leading to excess growth, nutrient storage and later disease [3], [4]. Still, “thrifty genes” [5] common to different species were not found and the “thrifty phenotype” framework remains hypothetical.

Emerging evidence from both human and animal studies suggests that programming may initiate very early in development, even during the periconceptional period. For example, the preimplantation embryo may respond to poor maternal diet, and indeed other conditions such as in vitro culture, by alterations leading to adult cardiometabolic disease [6], [7]. It has also been shown in animal models that maternal malnutrition can introduce changes in fetal DNA methylation and histone modifications at various genes, with some of these changes also observed postnatally [8], [9]. Moreover, the Dutch Famine cohort of individuals who were exposed to poor nutrition during the periconceptional period, more than sixty years later revealed hypomethylation of DNA at the imprinted IGF2 gene compared to a control group [10]. Further, maternal nutritional status at conception in Gambian women causes persistent and systemic epigenetic changes at metastable epialleles recognized postnatally [11]. These findings support the concept that intrauterine environment from before implantation can alter epigenetic marks that persist to adulthood [12].

Implicit within the thrifty phenotype hypothesis is that coordinated growth regulation for the whole organism in response to maternal dietary restriction will require a biosynthetic mechanism that is influential across diverse somatic tissues and species rather than one targeting individual genes. Indeed, we have shown that poor maternal nutrition in pigs and sheep caused global downregulation of gene transcription in fetal tissues [13].

Here we use a mouse model to investigate global transcription changes triggered by maternal protein restriction in fetal and adult offspring tissues. Our model comprises maternal isocaloric low protein diet (9% casein) fed either for the duration of pregnancy (LPD) or specifically for the preimplantation period only (Emb-LPD) with control normal protein diet (NPD, 18% casein) during the remainder of pregnancy and standard chow diet for all groups postnatally. In this model, both LPD and Emb-LPD lead to increased adult cardiometabolic disease and where neonatal weight is positively correlated with later adult disease risk [7], [14]. We focus on synthesis of the major cellular RNA species, ribosomal RNA, which consumes more than half of the cellular transcription resources, and is greatly influenced by nutrient availability. Collectively, our results indicate that maternal diet modulates the rate of rDNA transcription in the offspring. Critically, this mechanism is biphasic, with reduced rDNA transcription occurring during dietary challenge and enhanced (beyond control) transcription after release, and is activated from before embryo implantation. Moreover, we identify a role for the polymerase I–associated factor, Rrn3 [15], [16], in mediating the effect of maternal diet on rDNA transcription. We propose a novel developmental mechanism, activated before implantation, that fine tunes ribosome biogenesis. We also speculate that the pattern of rDNA expression during nutritional challenge and after release fulfills the requirements of a “thrifty gene” [5].

## 2 Materials and methods

### 2.1 Mouse model of maternal protein restriction

MF1 mice were bred in-house (University of Southampton Biomedical Research Facility) on a 0700–1900 light cycle with standard chow, under UK Home Office license and local ethics approval. Virgin females (7–8.5 weeks) were mated naturally overnight with MF1 males and plug positive females were housed individually the following morning and assigned randomly to either normal protein diet (18% casein, NPD) or isocaloric low protein diet (9% casein, LPD), the latter either for the gestation period or changed at embryonic day 3.5 (E3.5) to NPD for the remainder of gestation (Emb-LPD). Postnatal diet was standard chow in all groups. Diet composition has been described elsewhere [14], [17]. One animal per litter was used in experiments, n ≥ 6 per diet/age group (n = 12 for 17 dpc kidneys), 50% males and 50% females. Diet induced changes in cellular RNA content and rDNA methylation were not gender specific.

### 2.2 Per-cell RNA levels

To estimate RNA level per cell, we assumed that the number of cells in a tissue fragment is proportional to the DNA content, providing a simple way to normalize RNA levels: RNA/(cell number) ≈ RNA/DNA [18]. Nucleic acids, RNA and DNA, were simultaneously purified from fragments of fetal (17 dpc) and adult (6 mo) tissues by using Trizol reagent (Invitrogen, Gaithersburg, MD, USA); purified nucleic acids were dissolved in equal volumes of water, and OD260 was measured in each preparation by using spectrophotometer. Total RNA content per cell was estimated as the ratio of nucleic acid concentrations, [RNA]/[DNA].

### 2.3 Cell culture

Human kidney cell line HEK293 was used in these studies. Cells were grown in DMEM supplemented with glutamine, penicillin, streptomycin, and 10% fetal bovine serum. RNA purification, reverse transcription, Western blot analysis, and MeDIP assays were done as described below for kidney tissue. Transfections were done in 6-well plates using 0.5 μg of human RRN3 expression plasmid (HsCD00443164, DNASU) mixed with 2.5 μl of Lipofectamine 2000 reagent (Invitrogen, Gaithersburg, MD, USA) per well. After 48 h, transfected cells were analyzed using the same approaches described below for mouse tissues. Empty vector was used in control transfections. pEGFP plasmid was used in parallel to estimate transfection efficiency, which varied 60–70% between experiments.

### 2.4 Reverse transcription

For individual transcript analysis, RNA purified from animal tissues was treated with RNase-free DNase I, (Epicentre Technologies, Madison, WI) 1 U/10 μg of RNA for 15 min at 37 °C, deproteinized with phenol/chloroform mixture and precipitated with ethanol. One microgram of DNA-free RNA was reverse transcribed by Superscript III (100 U/reaction; Invitrogen, Gaithersburg, MD, USA) with random hexanucleotide primer mixture (1 μM) in 10 μl final volume for 1 h at 42 °C. Reaction was stopped by mixing with 90 μl TE (Tris–HCl 10 mM, pH 8.0, EDTA 1 mM) and incubation at 95 °C for 5 min. RT mixtures were further diluted ten times with TE and analyzed by real time PCR with gene-specific sets of primers. To normalize transcript levels, RT qPCR signal for each mRNA was divided by a control transcript signal. Two mRNAs, *Cypa* and *Lamc1*, were used as no change controls because these transcripts did not change in response to diet and showed no associations with gender.

### 2.5 DNA methylation analysis, MeDIP, and real time qPCR

1 μg of DNA purified from tissues was diluted to 0.5 ml with TE buffer, treated with ultrasound for 30 s (Bronson sonifier, equipped with a microtip), precipitated with ethanol, washed once with 70% ethanol, dried, and dissolved in 10 μl of TE buffer. Before immunoprecipitation (IP), DNA samples were boiled for 5 min, and chilled on ice. 0.5 μg of DNA was used in one IP reaction. MeDIP was done in 96-well plates as described [19], [20]. Monoclonal antibodies to 5mC (Mouse clone 33D3, Aviva, San Diego, CA, USA), 0.3 μg per IP reaction, were used. Mock IP was done without added antibodies. qPCR analysis of precipitated DNA was done with gene-specific primers. 10% of the amount of input DNA used in IPs was analyzed in parallel by qPCR to estimate efficiency of IP. Results were presented as percent of input precipitated by antibodies.

The reaction mixture contained 2.5 μl 2X SYBR Green PCR master mix (SensiMix, Bioline, UK), 2 μl DNA template and 0.25 μl primers (10 μM each) in 5 μl final volume in 384-Well Optical Reaction Plates (Applied Biosystems, Thermo Scientific, Waltham, MA, USA). Amplification (three steps, 40 cycles), data acquisition and analysis were carried out using the 7900HT Real Time PCR system and SDS Enterprise Database software (Applied Biosystems). All PCR reactions were done in triplicates. Standard dilutions of mouse genomic DNA (10, 1, 0.1, and 0.01 ng/μl) were included in each PCR run.

DNA methylation density at a DNA site was calculated as detailed in [19]. Results of MeDIP-qPCR analyses are presented as a percent of input DNA precipitated by 5mC antibodies. Results of MeDIP and gene expression analyses were evaluated statistically by using analysis of variance with maternal diet (LPD and Emb-LPD vs NPD) as experimental factor. Sequences of primers used in these studies are available upon request.

### 2.6 Chromatin immuno-precipitation, ChIP

ChIP was done in 96-well plates as previously described [19]. Antibodies to RNA-polymerase I were used (anti-POLRID, Pierce, Thermo Scientific, Waltham, MA, USA). Mock IP was done without added antibodies. Precipitated DNA was purified in 100 μl final volume. Input DNA was purified from 10% of the amount of tissue extract used in IPs. ChIP assays were repeated at least three times.

PCR analysis of ChIP DNA was done in triplicate as described above for MeDIP. Standard dilutions of mouse (or human, when appropriate) genomic DNA were included in each PCR run. ChIP data were expressed as a fraction of input DNA and normalized to NPD levels. Results of ChIP experiments were evaluated statistically by using analysis of variance with diet as experimental factor. Sequences of primers used in these studies are available upon request.

### 2.7 Western blot analysis

Whole cell extracts were purified from kidney tissue fragments as described in [21]. Protein concentration was measured using Bradford method (Thermo Scientific, Waltham, MA, USA). Equal amounts of protein were loaded on 10% SDS gels. After electrophoresis, proteins were transferred from gels on PVDF membranes. Membranes were blocked with TBST buffer (Tris–HCl, pH 7.5, 10 mM; NaCl, 150 mM; Tween 20, 0.05%) containing 5% BSA, and incubated with primary antibodies to either RRN3 (Thermo Scientific, Waltham, MA, USA) or β-actin proteins (Sigma, St. Louis, MO, USA) in TBST/1% BSA buffer for 1 h at room temperature, or overnight at 4 °C. After washes, secondary antibodies conjugated to alkaline phosphatase (Bio Rad, Hercules, CA, USA) were added for 1 h at room temperature. Membranes were washed with TBST and developed with BCIP/NBT substrate. Protein band intensities were measured by densitometry of membrane images using ImageJ64 software (National Institutes of Health, Bethesda, MD, USA).

### 2.8 Statistical analysis

All data were assessed for normality using the Shapiro–Wilk test. Tissues from only a single fetal or adult offspring per litter were analyzed to avoid confounding maternal origin effects. To account for gestational litter size, body weight and offspring sex interactions, RNA/DNA data were subjected to a univariate analysis of variance with post hoc Bonferroni's multiple comparisons test (SPSS version 21). MeDIP, ChIP, and RT PCR data were analyzed using ANOVA. Significance was taken at P < 0.05.

## 3 Results

### 3.1 Maternal diet modulates total RNA content per cell in offspring

**Fig. 1:**
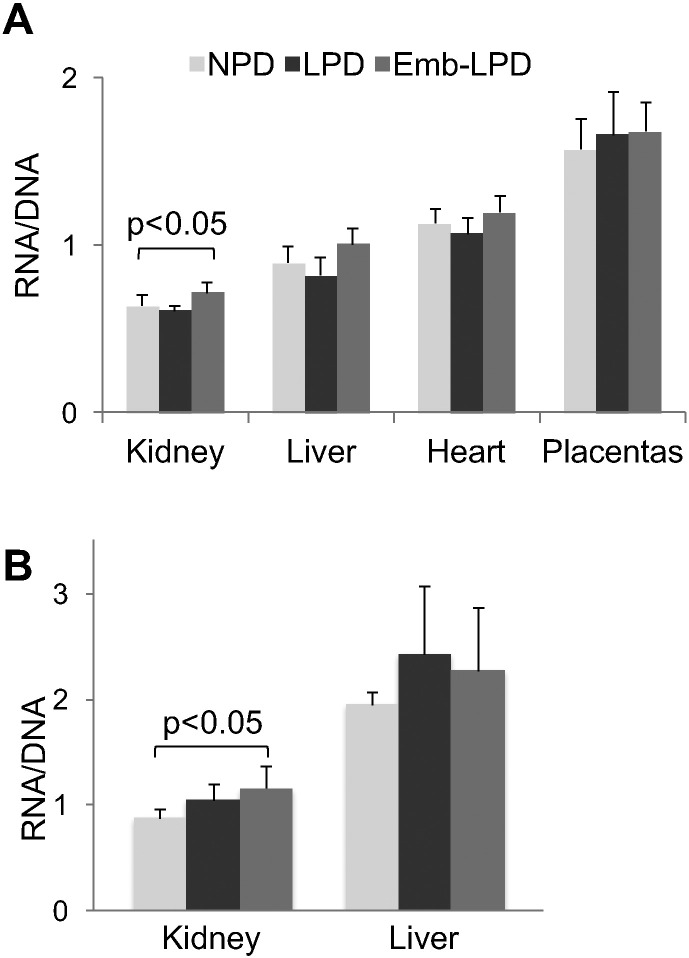
Maternal diet-induced changes in per cell RNA content in fetal and adult offspring tissues. RNA and DNA were simultaneously purified from 17 dpc fetal (A) and 6 months old (6 mo) adult (B) tissue fragments, dissolved in equal volumes of water, optical density was measured at 260 nm, and concentrations were calculated. Graphs represent RNA-to-DNA concentration ratios. Mean values are shown, ± SD, n ≥ 6.

Tissue samples extracted from 17 dpc fetuses and 6 mo adults following maternal LPD (LPD throughout gestation), Emb-LPD (LPD for preimplantation period only) and NPD treatments were used for simultaneous isolation of total RNA and DNA. To estimate changes in RNA per cell content associated with maternal diet, RNA:DNA ratios were measured [13] (Fig. 1). Examined 17 dpc fetal tissues (kidneys, livers, and hearts) show the same pattern, a consistent decrease in RNA:DNA ratio in the LPD group, but an increase in the Emb-LPD group compared to NPD controls (Fig. 1A). Combined data from fetal kidneys, livers and hearts show this pattern to be statistically significant, revealing systemic response (Additional data file Figure S1). In contrast, data obtained in placentas reveal no difference between the diet groups (Fig. 1A).

Analysis of livers and kidneys in adult offspring following both LPD and Emb-LPD treatments showed increased RNA:DNA ratios compared with NPD controls (Fig. 1B; see also Additional data file Figure S1). We conclude that during exposure to LPD, cellular RNA content is decreased, while switching to normal diet, either before (Fig. 1A, Emb-LPD) or after (Fig. 1B, LPD) birth, increases the amount of RNA per cell beyond that of the unchallenged NPD group. Ribosomal RNA, rRNA, accounts for ~ 80% of cellular RNA species in living cells. Therefore, we next estimated diet-induced changes in rDNA transcription rates and potential underlying epigenetic regulatory processes.

### 3.2 Maternal diet regulates rDNA transcription levels in offspring

**Fig. 2:**
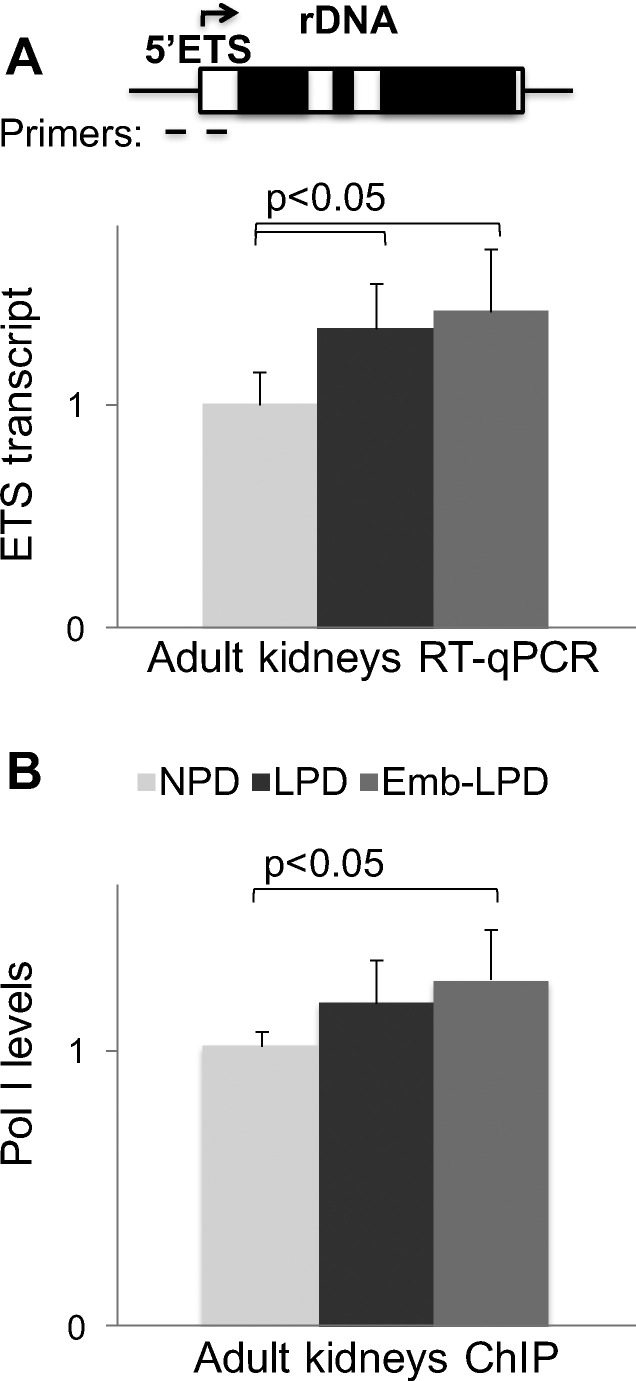
Changes in rDNA transcription induced by maternal diet in adult kidneys. RT qPCR analysis of pre-rRNA transcript levels (A). RNA from adult kidneys was reverse transcribed and analyzed by qPCR with primers to ETS shown in *upper panel*. ETS PCR signal was normalized to two control transcripts, *Cypa* and *Lamc1*, data were combined, adjusted to NPD levels, and presented as mean ± SD, n = 6. (B) Matrix ChIP analysis of Pol I recruitment to rDNA. Kidney fragments from adult NPD, LPD, and Emb-LPD kidneys were fixed with formaldehyde and used to prepare chromatin fractions. Equal amounts of samples were precipitated with POLR1D antibodies, isolated DNA was analyzed by qPCR with primers to rDNA core promoter (100 bp upstream of transcription start site, as shown in *upper panel* (A)). ChIP data were normalized to input DNA signals, and adjusted to NPD levels, mean ± SD shown, n = 6.

To test the hypothesis that rDNA transcription in the adult offspring was altered by maternal diet, we examined levels of pre-rRNA transcript with primers to 5′ external transcribed sequence, ETS (Fig. 2A *upper panel*). ETS is not present in mature transcript and can be used to estimate rDNA transcription rates [22], assuming that rRNA processing is not altered [23], [24]. Fig. 2A shows that ETS levels were higher in LPD and Emb-LPD kidneys compared to NPD. As an alternative way to estimate transcription intensity, next we used ChIP assay to assess occupancy of RNA polymerase I at rDNA repeats in adult kidneys. Note, that ChIP qPCR signal is divided by the input DNA qPCR signal, which normalizes for inter-individual variation in rDNA copy number per genome. Results of ChIP analysis demonstrate that Pol I density at rDNA is significantly higher in Emb-LPD than in NPD samples (Fig. 2B). Together with increased ETS levels (Fig. 2A) these data support upregulation of rDNA transcription rather than changes in rRNA stability.

DNA methylation is an epigenetic marker enriched at transcriptionally silenced genes, but low at expressed genes [25], [26]. There are several approaches to estimate changes in the level of this modification at a genomic site of interest, including bisulfate sequencing [26] and Methylated DNA Immuno-Precipitation assay, MeDIP [27]. MeDIP is done with antibodies specific to 5-methyl cytosine, 5mC, which we have recently modified to use in 96-well plates where antibodies are attached to the well walls [28]. This modification of the protocol simplified the assay and substantially improved data reproducibility.

**Fig. 3:**
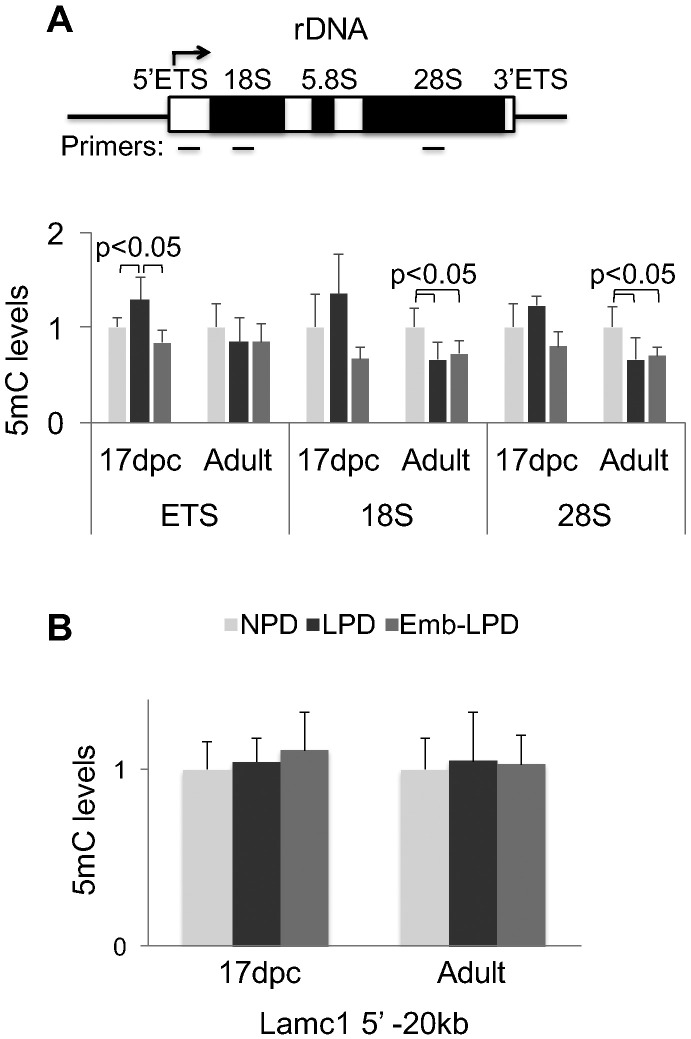
Diet-induced changes in the level of DNA methylation at rDNA locus. MeDIP assay was used to examine 5mC levels at rDNA (A) and a control genomic site (Lamc1 (B)) in 17 dpc fetal and adult kidneys. *Upper panel* (A) is a cartoon of rDNA transcription unit. Arrow marks transcription start site, open boxes depict 5′ and 3′ ETS external transcribed sequences and internal transcribed sequences not present in mature rDNA, closed boxes represent 18S, 5.8S, and 28S transcripts. Positions of PCR primers are shown below rDNA locus. MeDIP analysis was done with DNA purified from either 17 dpc or adult kidney segments, MeDIP DNA and input DNA were analyzed by qPCR with rDNA primers shown in *upper panel* (A), and primers to a genomic locus upstream of Lamc1 transcription start site (− 20 kb) (B). MeDIP data were normalized to input and adjusted to NPD levels, mean ± SD shown, n = 6.

Results of MeDIP qPCR analyses of DNA from kidneys are shown in Fig. 3. In 17 dpc fetal samples, the level of rDNA methylation was increased in LPD but not Emb-LPD compared to NPD animals (Fig. 3A). In 6 mo adults, LPD and Emb-LPD kidneys had reduced rDNA methylation compared to NPD (Fig. 3B). These changes in the level of 5mC at rDNA therefore inversely correlate with changes in RNA per cell content (compare to Fig. 1A and B). Methylation levels of a control genomic site, a silenced region upstream of *Lamc1* gene transcription start site (− 20 kb) [29], were not altered by maternal diet in both age groups (Fig. 3B), supporting the specificity of changes at rDNA loci. Similar alterations in rDNA methylation were also found in livers from the same animal groups (Additional data file Figure S1). These observations indicate that cellular RNA content and rDNA methylation are linked in animal tissues and that transcription changes associated with rDNA methylation at least in part account for changes in per cell RNA content induced by maternal diet.

### 3.3 Changes in rDNA transcription are matched by expression pattern of rDNA transcription factor, Rrn3

**Fig. 4:**
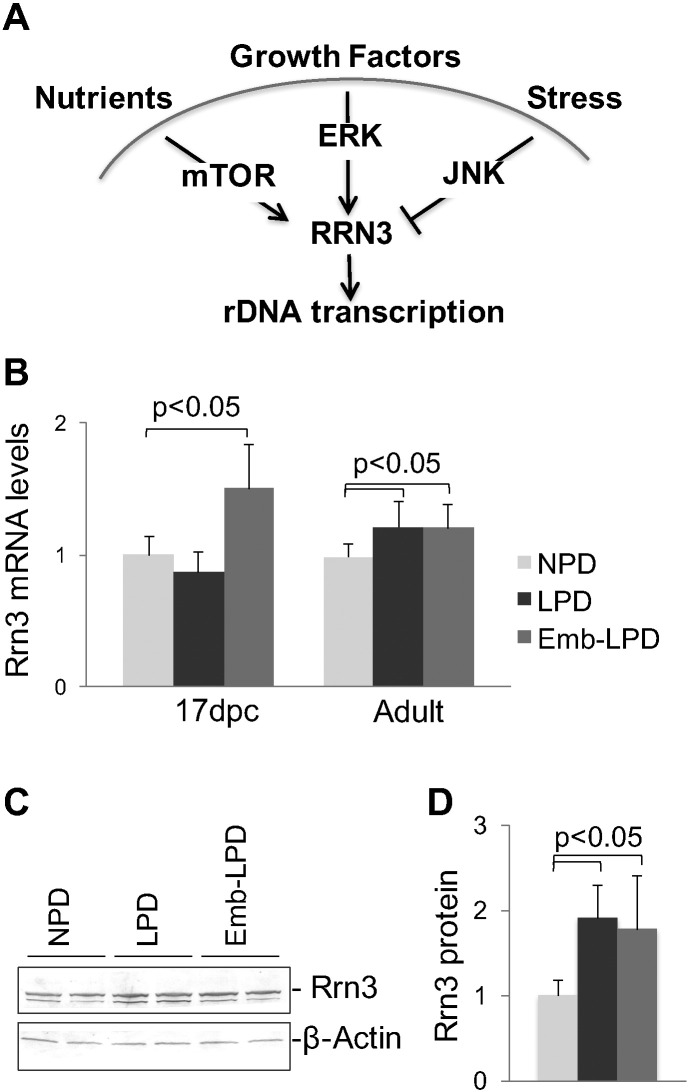
The effect of maternal diet on expression levels of Rrn3 transcription factor in the offspring. (A) A cartoon illustrating the role of Rrn3 in mediating extracellular signals to rDNA transcription by Pol I. (B) RT PCR analysis of *Rrn3* transcript levels in 17 dpc and adult kidneys. RNA purified from kidneys was treated with DNase, reverse transcribed with random hexamer primers, and analyzed by qPCR with two different pairs of primers to the last exon of *Rrn3* gene. Transcript levels were normalized to two control transcripts, *Cypa* and *Lamc1*, data were combined, adjusted to NPD levels, and presented as mean ± SD. (C) Western blot analysis of Rrn3 protein levels in adult kidneys. Extracts from kidneys (male), 50 μg total protein per lane, were electrophoresed in SDS gels, transferred to PVDF membrane and probed consecutively with antibodies to Rrn3 and β-actin proteins. After incubation with secondary antibody conjugated with alkaline phosphatase, membranes were developed with NBT/BCIP substrate. Representative membrane is shown. (D) Densitometry of Rrn3 band intensities. Western blot membranes were scanned and Rrn3 and β-actin band densities were measured using ImageJ64 software. Graph shows Rrn3/β-actin ratios adjusted to NPD levels, mean ± SD, n = 6.

Diet-induced changes in rDNA transcription are most likely mediated by transcription factors that transmit nutritional signals to the Pol I machinery. Most of the factors that drive rDNA transcription are well studied. While Pol I is very abundant, it cannot initiate transcription without transcription factors such as *Ubf*, *Sl1* and *Rrn3*, where complex formation is regulated by kinase signaling pathways providing a link between nutrient availability and rDNA transcription [15], [22]. Rrn3 is positively controlled by external signals, including nutrients and growth factors, while negatively regulated by stress [15], [30] (Fig. 4A). Moreover, relevant to our model, it has been shown that Rrn3 activity limits rDNA transcription in amino acid starved cells [30]. Deficient amino acids in uterine fluid following Emb-LPD has been proposed to activate altered developmental programming of blastocysts [31]. Moreover, we have recently found that in vitro embryo culture at deficient amino acid concentrations followed by transfer is sufficient to recreate the postnatal growth and cardiometabolic phenotype found in Emb-LPD offspring (unpublished results). Therefore, we set out to examine changes in the level of *Rrn3* gene expression in response to maternal diet. These experiments were conducted in kidneys, tissue that shows consistently significant changes in all previous assays (Fig. 1, Fig. 2, Fig. 3).

RT PCR analysis of RNA purified from kidneys was performed with two different primer pairs to *Rrn3* transcript, and PCR signals were normalized to two different control transcripts, *Cypa* and *Lamc1*, that are abundantly expressed in kidneys and other tissues and do not respond to diet. RT PCR data are presented in Fig. 4B. Fetal LPD kidneys show decreased whereas Emb-LPD kidneys significantly increased *Rrn3* mRNA levels compared to NPD animals. In adults, both LPD and Emb-LPD kidneys reveal elevated levels of the transcript compared to control animals. We also found similar changes in *Rrn3* transcript levels in adult livers from the same animal groups (Supplemental Figure S3). These findings are further supported by results of Western blot analysis of Rrn3 protein changes in adult kidneys (Fig. 4C, D, males and females combined. Gender specific data are shown in Additional data file Figure S4). Rrn3 protein levels were significantly higher in LPD and Emb-LPD tissue extracts compared to NPD samples.

### 3.4 Rrn3 protein regulates per cell RNA content, rDNA methylation levels and rDNA transcription rates in vitro

**Fig. 5:**
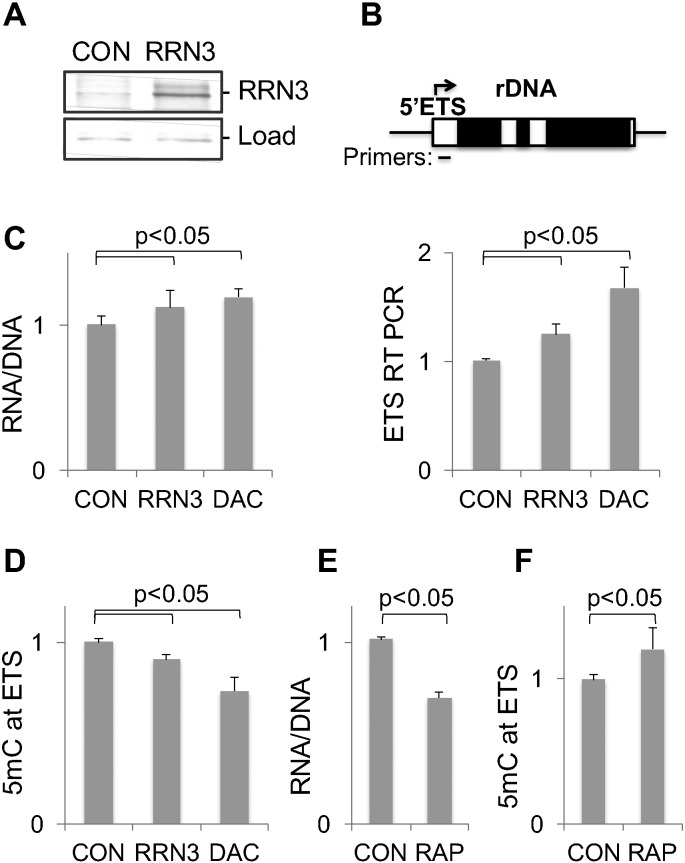
The effect of RRN3 gene over-expression on rDNA transcription. (A) HEK293 were transfected with RRN3 plasmid for two days (*RRN3*), whole cell proteins were extracted and analyzed by Western method with RRN3 antibody. *CON*, cells transfected with empty plasmid. *Load*, a fragment of Ponceau S stained membrane. (B) Upper panel, gene position of primers used in qPCR. Graph shows results of RT qPCR analysis of RNA purified from transfected cells and from cells treated with 5 μM DAC for two days. ETS data were normalized to Lamc1 transcript levels. (C) RNA/DNA ratios were measured in the same cells. (D) MeDIP analysis of DNA purified from the same cells. The effect of rapamycin treatment (50 nM, 48 h) on (E) RNA/DNA ratio, and (F) DNA methylation in HEK293 cells. All data were normalized to CON levels, bars represent mean ± SD from at least three independent experiments.

Upregulation of Rrn3 in LPD/Emb-LPD adults suggests that this factor is involved in diet-induced changes in rDNA transcription. In fact, it has been shown that overexpression (OE) of Rrn3 protein in cell culture elevated rDNA transcription, however other published data support an alternative scenario, where cellular levels of Rrn3 protein are less important for rDNA transcription than its phosphorylation state [15], [22], [30]. To re-examine this issue, we overexpressed human RRN3 protein from a plasmid in human kidney cell line HEK293. Results of these experiments demonstrate that RRN3 protein OE (Fig. 5A) increases cellular levels of 5′ external transcribed sequence, ETS (Fig. 5B), supporting upregulated rDNA transcription, and significantly increases cellular RNA content (i.e. RNA/DNA ratio) (Fig. 5C). Importantly, RRN3 OE also causes significant decrease in rDNA methylation levels (Fig. 5D), supporting inverse link between rDNA transcription and methylation observed in vivo (Fig. 3). Functional significance of rDNA methylation changes is demonstrated in cells treated with DNA methylation inhibitor 5-Aza-2′-deoxycytidine (DAC) where rDNA demethylation is associated with increases in ETS transcript levels and RNA/DNA ratio (Fig. 5B–D).

To suppress RRN3 activity we treated cells with rapamycin, a potent inhibitor of rDNA transcription [32]. Although rapamycin has many effects on cellular processes, several observations identified RRN3 (along with other candidates [33]) as one of the key targets of rapamycin in rDNA transcription: i) RRN3 factor is sufficient to restore rDNA transcription in extracts prepared from rapamycin-treated mammalian cells [34], ii) rapamycin treatment decreases nuclear concentration of RRN3 protein [34], and iii) Rrn3 overexpression makes rDNA transcription resistant to rapamycin in vivo (in Drosophila [35]). In agreement with RRN3 OE data, cells treated with rapamycin reveal a decrease in RNA/DNA ratio and an increase in rDNA methylation levels (Fig. 5E and F, respectively). These observations define RRN3 as one of the key candidate factors that mediate the effect of poor intrauterine environment on rDNA transcription and methylation in adult offspring. However, the effect of RRN3 OE on rDNA transcription in vitro is smaller than upregulation of rDNA in LPD and Emb-LPD adult offspring, indicating that other factors may also play a role in vivo, including kinases that activate RRN3 [36], UBF [33] and SL1 [22], [34].

## 4 Discussion

We found that dietary restrictions during pregnancy in mice induce changes in cellular RNA content in fetal tissues. These observations are consistent with previous studies showing that maternal protein restriction in pigs and placental insufficiency in sheep [13] both decrease per cell RNA content in fetal tissues during the challenge. Thus global cellular transcription rates in fetal organs are tightly regulated with nutrient availability across different mammalian species. This study extends previous observations by showing that the ‘memory’ of poor nutrition in utero is preserved after birth and results in excessive rDNA transcription rates when animals have access to plentiful nutrients. rDNA transcription and ribosome biogenesis consume more than half of cellular energy resources [37], playing a major role in the control of nutrient utilization balance in the cell. As such, rDNA transcription might be one of the primary targets of developmental programing of transcription in response to poor intrauterine nutrient environment.

Interestingly, these observations identify rDNA as a plausible candidate for a “thrifty gene” suggested by Neel half a century ago [5]. Trying to explain high incidence of type 2 diabetes in modern societies, he proposed existence of “thrifty genes” that were evolutionary helpful to survive famines, but became disadvantageous in current environment. However, no “thrifty genes” were discovered so far. We found that rDNA transcription switches to economy (thrifty) mode during nutritional challenge, whereas after release from challenge, rDNA transcription exceeds control levels, which may cause accelerated unbalanced growth. Therefore, rDNA behaves as a thrifty gene involved in nutrient utilization control, which is tuned by nutrient availability in utero.

We found that characteristics that determine adult rDNA transcription rates, including DNA methylation profile, and Rrn3 protein levels, are set during the preimplantation period of mouse development. During this period, paternal and maternal genomes undergo global changes in DNA methylation patterns [38], [39], [40], [41], which start with active (paternal) and replication-dependent passive (maternal) DNA demethylation, followed by re-establishment of methylation patterns. We found that compared to the control NPD group, in Emb-LPD, rDNA methylation was decreased and rDNA transcription increased together with Rrn3 expression during both fetal and adult life, demonstrating the prolonged legacy of the preimplantation nutrient experience. Since rDNA expression profile in LPD fetal tissues was distinct and opposite from Emb-LPD, it demonstrates that rDNA transcription rate is not pre-programmed (pre-set) by maternal diet during the preimplantation period, but rather is a dynamic response to dietary exposures, modified by signals from upstream regulators, including Rrn3 transcription factor. Systems that regulate Rrn3 activity remain to be discovered.

Several studies have shown that cellular RNA content closely mirrors cell size [42], [43], [44], therefore enhanced rDNA transcription observed in offspring of malnourished dams is most likely a reflection of accelerated cell growth [45]. Our data in this respect fit the growth phenotype of Emb-LPD offspring where enhanced growth relative to NPD is evident [14]. Diet induced changes in cellular RNA content in fetal tissues were not matched by similar changes in placentas (Fig. 1A). This distinction between embryonic and extra-embryonic lineages is also evident in our past work where the Emb-LPD preimplantation embryo activates compensatory cellular responses within the placental lineages, coordinated by cellular and epigenetic mechanisms, to improve the efficiency of maternal nutrient delivery [14], [31], [46], [47]. As a consequence, we find the preimplantation embryo is able to program lineage-specific responses to malnutrition, to promote maternal nutrient supply (extra-embryonic) and to adjust growth to nutrient availability (embryonic).

The capacity to fine-tune ribosomal biosynthesis across the lifespan from preimplantation experience onwards has important implications. There are obvious evolutionary benefits for growth to be regulated by nutrient availability, limited during austerity but stimulated by later abundance, an opportunistic mechanism to support competitive reproductive fitness. However, the combination of restricted gestational growth in response to maternal malnutrition and enhanced compensatory growth postnatally is the hallmark of adverse developmental programming [1], [2], [3], [4]. Adult Emb-LPD female mice display these characteristics including overgrowth, adiposity and increased cardiometabolic disease risk relative to NPD offspring [14], [48], which may reflect at least in part the maladaptive consequences of the programmed stimulation of rDNA transcription and ribosome biogenesis. As well as cardiometabolic disease, birth weight is also associated with risk of several cancers in later life [49] such as testicular [50], [51] and mammary [52] cancers. Although these events coincided with altered expression of mitogen and tumor suppression factors [53], [54], the involvement of ribosome biogenesis has not been explored. In addition, given the periconceptional origin of dietary programming of rDNA expression, other environmental conditions not directly nutritional, may also influence rDNA. Epidemiological screening of IVF children indicates increased prevalence of developmental changes including low birth weight, increased growth trajectory during infancy, and cardiometabolic disease risk [55], [56], [57]. Although ribosome biogenesis has not been explored in IVF children, it is noteworthy that rRNA and Pol I-dependent transcripts are sensitive to IVF and nuclear transfer in mouse embryos [58].

## 5 Conclusion

We have identified a mechanism active from the preimplantation period whereby maternal dietary nutrition during gestation has a long term effect on systemic rDNA transcription. This mechanism activated by nutritional challenge increases rDNA expression beyond that of controls after release from challenge and persists across the lifespan. We provide in vivo and in vitro evidence that rDNA methylation and Pol I transcription factor Rrn3 mediate the maternal diet-induced changes in rDNA transcription rates. We propose that such regulation of rDNA expression offers a molecular explanation of the thrifty phenotype concept of globally restricted growth during nutrient challenge and excess growth after release from challenge.

## Competing interests

The authors declare that they have no competing interests.

## Authors' contribution

OD conceived these studies; OD, KB and TPF designed experiments; OD, ESL, CS, AJW, and DM acquired data, all authors participated in data analysis and interpretation; OD drafted the manuscript and all authors critically revised it. All authors agreed to be accountable for all aspects of the work in ensuring that questions related to the accuracy or integrity of any part of the work are appropriately investigated and resolved.

## Transparency document

Transparency document. Image 2

## Appendix A Supplementary data

Supplementary figures. Image 3
