# Supplementary material for: Regulation of ribosomal RNA expression across the lifespan is fine-tuned by maternal diet before implantation
Source: Biochim Biophys Acta. 2016 Jul;1859(7):906–13. doi: 10.1016/j.bbagrm.2016.04.001 (PMC4914606; doi:10.1016/j.bbagrm.2016.04.001)
Supplement: Supplementary file 2 — Supplementary figures. [file mmc2.pdf]

Supplementary Data

Regulation of ribosomal RNA expression across the lifespan is fine-tuned by maternal diet before implantation

Oleg Denisenko[a], Emma S. Lucas[b], Congshan Sun[b], Adam J. Watkins[b], Daniel Mar[a], Karol Bomsztyk[a], and Tom P. Fleming[b]

From the [a]Department of Medicine, University of Washington, 850 Republican St., Room 242, Seattle, WA 98109, USA, and [b]Centre for Biological Sciences, University of Southampton, Mailpoint 840, Level D Lab & Path Block, Southampton General Hospital, Tremona Road, Southampton, SO16 6YD, UK

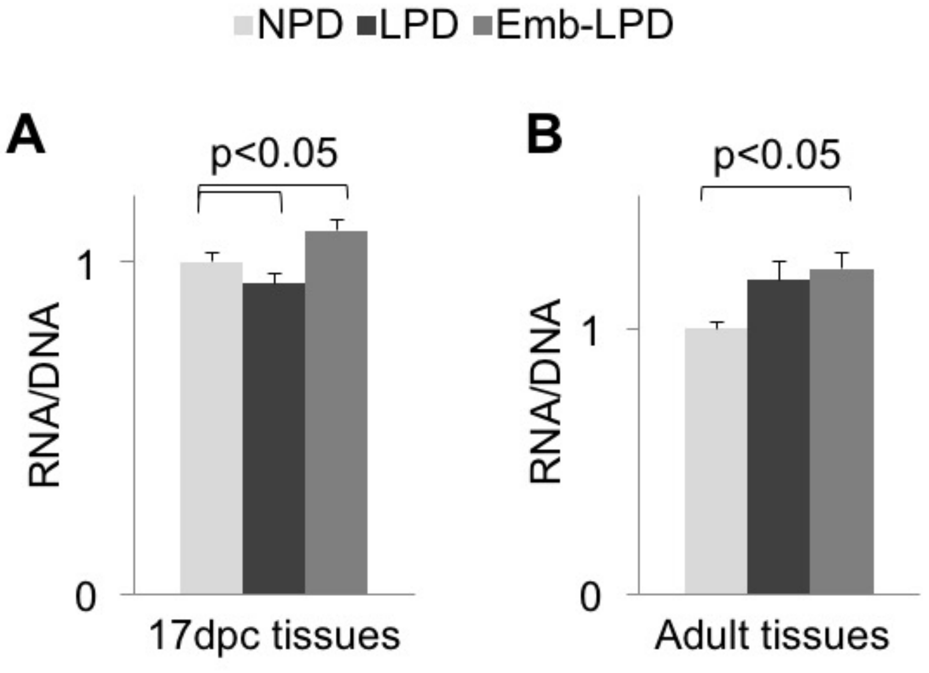

Figure S1. Maternal diet-induced changes in per cell RNA content in 17 dpc tissues (kidneys, livers, hearts) (**A**) adult (kidneys and livers) (**B**) combined. RNA and DNA were simultaneously purified from whole tissue fragments, dissolved in equal volumes of water, optical density was measured at 260 nm. Graphs represent RNA-to-DNA concentration ratios. Mean values are shown, ± SEM, n=6 per group.

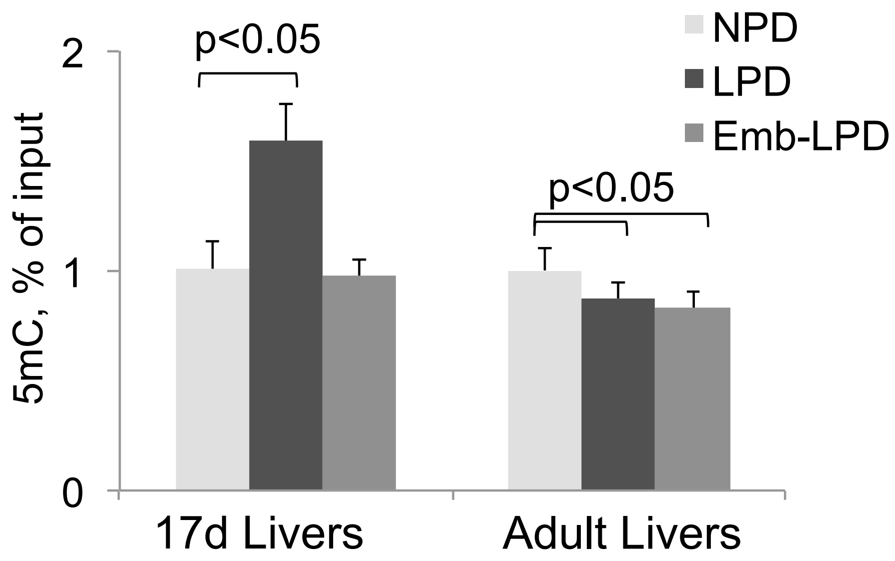

Figure S2. Diet-induced changes in the level of DNA methylation at rDNA locus in adult livers. DNA was purified from adult liver fragments, treated with ultrasound, made single stranded by boiling, and analyzed by MeDIP with 5mC antibodies. Immuno-precipitated DNA and input DNA were analyzed by qPCR with rDNA primers shown in Fig.3. MeDIP data are presented as percent of input adjusted to NPD levels, Mean ± SEM, n=6 per group. For each rDNA primer pair, data were normalized to NPD values.

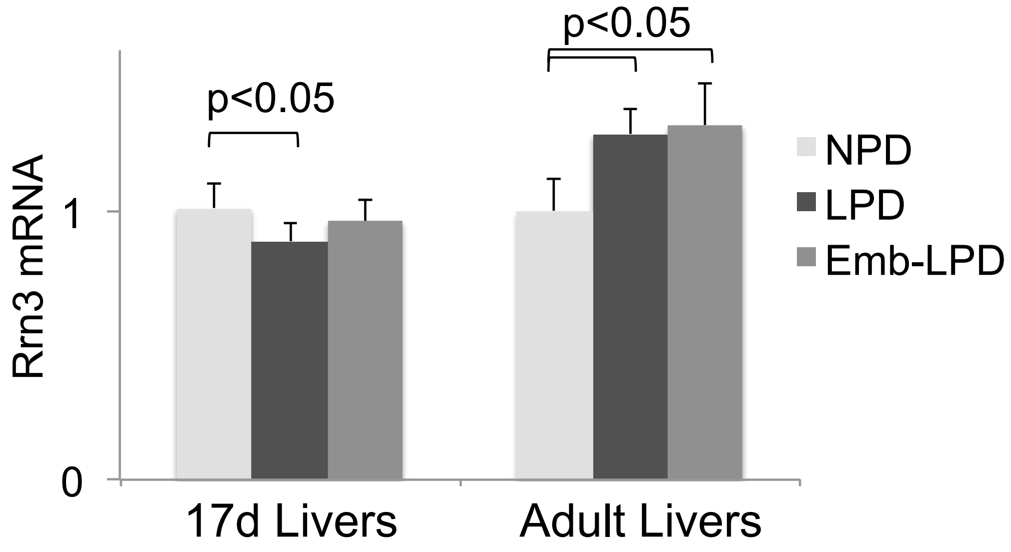

Figure S3. The effect of maternal diet on expression levels of Rrn3 mRNA in adult offspring. RT PCR analysis of *Rrn3* transcript levels. RNA purified from livers was treated with DNase, reverse transcribed with random hexamer primers, and analyzed by qPCR with two different pairs of primers to the last exon of *Rrn3* gene. Transcript levels were normalized to two control transcripts, *Cypa* and *Lamc1*, and adjusted to NPD levels. Data are presented as Mean ± SEM, n=6 per group.

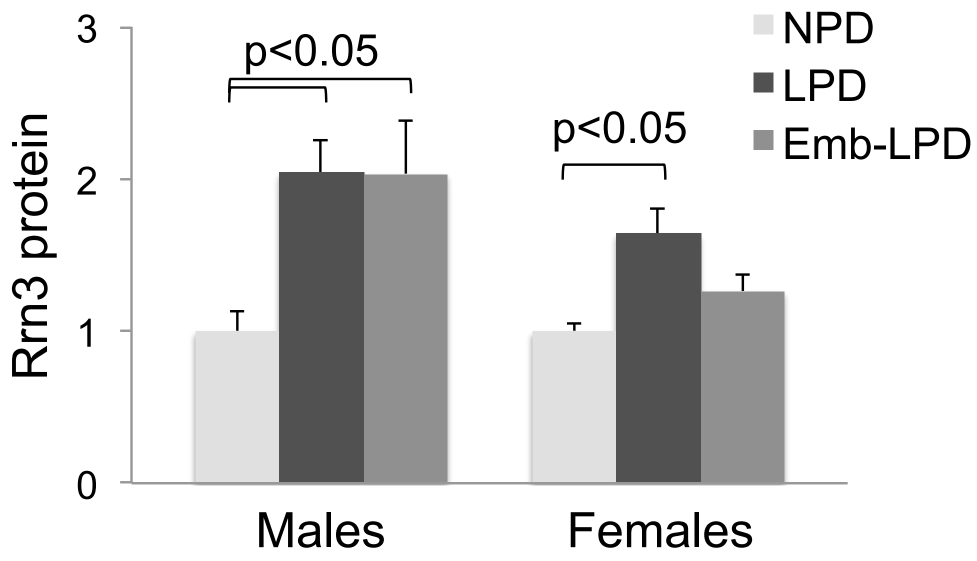

Figure S4. Western blot analysis of Rrn3 protein levels in male and female adult kidneys. Extracts from kidneys, 50 µg total protein per lane, were electrophoresed in SDS gels, transferred to PVDF membrane and probed consecutively with antibodies to Rrn3 and β–actin proteins. After incubation with secondary antibody conjugated with alkaline phosphatase, membranes were developed with NBT/BCIP substrate. Results of densitometry of Rrn3 band intensities are shown as Rrn3/β-actin ratios adjusted to NPD levels, Mean ± SD, n=3 males, and 3 females per diet group.
